# Biofilm-host immunometabolic crosstalk in *Staphylococcus aureus* orthopedic implant infection: mechanisms and translational constraints

**DOI:** 10.3389/fimmu.2026.1957346

**Published:** 2026-10-05

**Authors:** Jixin Chen, Qinxin Zhou, Dongdong Cao, Weijie Yu, Yongming Zhang, Jianliang Chen, Xiaodong Zheng, Feng Ye

**Affiliations:** 1 Department of Orthopaedic Surgery, Shaoxing Shangyu District Hospital of Traditional Chinese Medicine, Shaoxing, China; 2 Department of Orthopaedic Surgery, Shaoxing Hospital of Traditional Chinese Medicine Affiliated to Zhejiang Chinese Medical University, Shaoxing, China; 3 Department of Orthopaedic Surgery, Chongqing University Jiangjin Hospital, Chongqing, China; 4 Department of Orthopaedic Surgery, YunNan Provincial Hospital of Traditional Chinese Medicine, Kunming, China

**Keywords:** biofilm, immunometabolism, macrophages, myeloid-derived suppressor cells, neutrophils (PMNs), orthopedic implant infection, *Staphylococcus aureus*

## Abstract

Orthopedic implant biofilms are not only antibiotic-tolerant bacterial communities but metabolically active niches that reshape host immunity. Compared with planktonic infection, mature biofilms impose spatial and metabolic constraints that can uncouple leukocyte recruitment from sterilizing activity. Neutrophils can penetrate and phagocytose portions of Staphylococcus aureus biofilms, but clearance becomes incomplete as biofilms mature and may coexist with degranulation and neutrophil extracellular trap-associated tissue injury. Macrophages show impaired access and bactericidal programming, and biofilm-derived lactate can promote interleukin-10 (IL-10) through histone deacetylase 11 (HDAC11)/HDAC6-dependent epigenetic reprogramming in experimental systems. Human periprosthetic joint infection (PJI) tissues are enriched in granulocytic myeloid-derived suppressor cells (G-MDSCs), whereas the glycolysis/hypoxia-inducible factor 1-alpha (HIF-1alpha) phenotype and its contribution to persistence are supported primarily by murine PJI, with limited human transcriptomic corroboration. In chronic S. aureus infection models, MDSC-derived lactate can suppress CD4-positive (CD4+) T-cell glycolysis; conversely, immune pressure drives heterogeneous bacterial metabolic, stress-response, and virulence programs. We synthesize these interactions as bidirectional biofilm-host immunometabolic crosstalk linking persistent infection to bone-interface damage. Source control and antibiotics remain essential. Local, time-limited, dysfunction-directed host interventions are presented here as a hypothesis-generating translational framework rather than evidence-based clinical recommendations, because host-directed immunometabolic therapies for PJI remain preclinical.

## 1 Introduction

Orthopedic implant infection is frequently described as an antibiotic problem: microorganisms adhere to a foreign surface, develop a biofilm, and become tolerant to antimicrobial therapy (1). This account is correct but incomplete. Compared with planktonic bacteria, biofilm organisms are embedded within an extracellular matrix, display marked growth and metabolic heterogeneity, and experience gradients of oxygen, nutrients, pH, and metabolites (2–4). These features can restrict phagocyte access, alter the microbial signals encountered by leukocytes, and reshape local immune-cell metabolism. Planktonic infection more often presents broadly accessible microbial targets and an acute inflammatory challenge, whereas mature biofilm can sustain chronic, spatially constrained inflammation in which leukocyte recruitment is uncoupled from effective clearance. The paradox is therefore not immune absence but immune activity that is abundant, spatially misdirected, and functionally ineffective (2).

Importantly, a clinically relevant biofilm phenotype does not require permanent attachment to an implant. In infected synovial fluid, staphylococci can form non-adherent, free-floating biofilm-like aggregates surrounded by extracellular material, including host-derived fibrin; these aggregates share with attached biofilms reduced susceptibility to antimicrobials and phagocytic clearance (5, 6). *In vitro*, pre-formed free-floating Staphylococcus aureus aggregates can also generate attached biofilms under flow, suggesting that suspended and surface-associated states may coexist within an infected joint (7).

Recent reviews have comprehensively addressed skeletal infection immunity and S. aureus implant-biofilm immune dysfunction, including G-MDSC biology and immunometabolic mechanisms (2, 8, 9). The incremental focus of this Mini Review is therefore deliberately narrower. Rather than cataloguing immune-evasion mechanisms, we organize the evidence around bidirectional immunometabolic exchange: how biofilm architecture and metabolites reprogram neutrophils, macrophages, G-MDSCs, and T cells, and how leukocyte pressure and the local tissue niche reciprocally reshape bacterial physiology. We explicitly distinguish human observational evidence from mechanistic evidence obtained in animal and ex vivo models, connect these pathways to the bone-implant interface, and evaluate host-directed adjuncts through a risk-aware translational lens. Using S. aureus as the mechanistic anchor, we also consider attached and free-floating biofilm states while treating mechanisms derived from this organism as hypotheses to test across the broader microbiology of PJI. A conceptual overview of this framework is shown in **Figure 1**.

**Figure 1:**
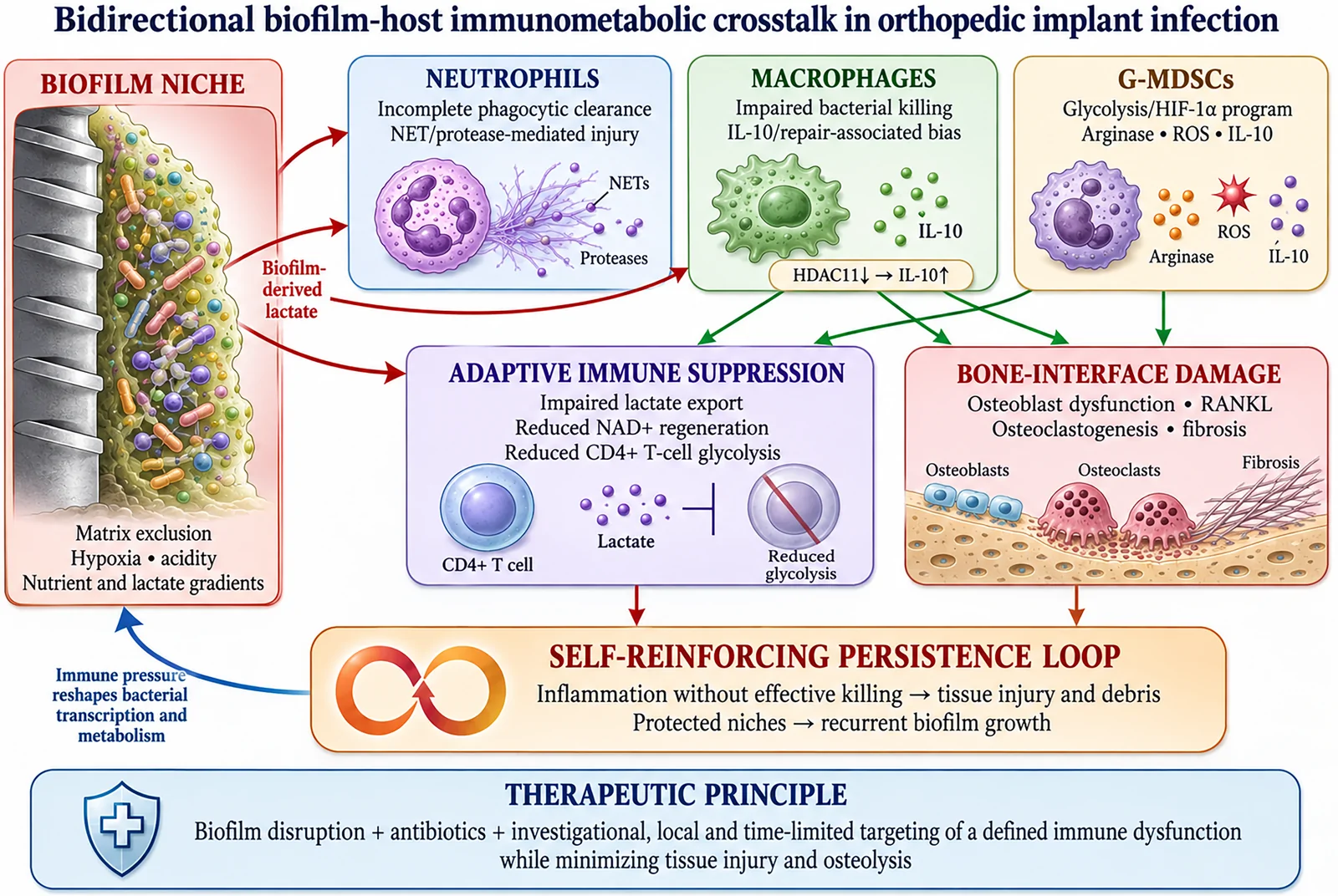
Conceptual model of bidirectional immunometabolic crosstalk during *Staphylococcus aureus* orthopedic implant biofilm infection. The biofilm matrix and local oxygen, nutrient, and metabolite gradients restrict immune access and shape leukocyte metabolism. Neutrophils may penetrate and phagocytose biofilm-associated bacteria, but clearance can remain incomplete as biofilms mature and may coexist with degranulation and NET formation. Macrophages show impaired access and bactericidal metabolic programming. In experimental systems, biofilm-derived lactate inhibits HDAC11, permits increased HDAC6 activity, increases histone H3 acetylation at the Il10 promoter, and enhances IL-10 production in macrophages and MDSCs. G-MDSC glycolysis/HIF-1alpha activity and lactate-associated metabolic suppression can impair adaptive immune function, while immune pressure reciprocally drives heterogeneous bacterial metabolic, stress-response, and virulence programs. These interactions contribute to osteoblast injury, RANKL-associated osteoclastogenesis, fibrosis, reduced perfusion, and a self-reinforcing persistence loop. Therapeutic elements shown in the schematic represent hypothesis-based investigational concepts rather than established clinical recommendations. The schematic was prepared specifically for this Mini Review and is not adapted from a previously published figure. Abbreviations: G-MDSC, granulocytic myeloid-derived suppressor cell; HDAC, histone deacetylase; HIF-1alpha, hypoxia-inducible factor 1-alpha; IL-10, interleukin-10; MDSC, myeloid-derived suppressor cell; NET, neutrophil extracellular trap; RANKL, receptor activator of nuclear factor-kappaB ligand.

## 2 Biofilm is a host-remodeling niche

The biofilm matrix is often portrayed as a passive diffusion barrier. In reality, it is a dynamic composite of microbial polymers and host-derived material. Bacterial extracellular polysaccharides, proteins, extracellular deoxyribonucleic acid (DNA), membrane-associated products, and lysed-cell debris can be interwoven with host proteins and inflammatory material. Gradients of oxygen, pH, nutrients, and metabolites generate bacterial subpopulations with different growth rates and stress responses (3, 4). This heterogeneity reduces antibiotic susceptibility, changes which microbial ligands are visible to immune cells, and creates local microenvironments in which immune-cell metabolism and bacterial metabolism compete for the same substrates.

Host incorporation is particularly important in the joint space. Within minutes of contact with synovial fluid, S. aureus can bind fibrinogen and fibronectin through surface adhesins, promoting macroscopic aggregation; fibrinolysis reduces aggregate formation and can increase antimicrobial susceptibility in experimental systems (6, 10). Clinical synovial-fluid aggregates have been shown to contain synovial fluid-derived fibrin (5). Hyaluronan can also be incorporated into the S. aureus biofilm matrix in implant-infection models, while albumin, fibrinogen, and hyaluronic acid influence the architecture and phenotype of floating synovial-fluid biofilms (11, 12). Neutrophil-derived extracellular DNA and proteins may further be deposited into the matrix during extracellular-trap responses (13, 14). These host components are therefore not merely contaminants: they can increase aggregate cohesion, alter porosity and diffusion, provide additional binding sites, and partially shield microbial structures from effective immune recognition.

A surface is therefore not required for a clinically relevant biofilm phenotype. Direct microscopy of infected synovial fluids has demonstrated Staphylococcus aureus and Staphylococcus lugdunensis as single cells mixed with biofilm-like clumps encased in exopolymeric material containing host fibrin (5). Synovial fluid can rapidly promote free-floating S. aureus aggregation while simultaneously reducing immediate attachment to several orthopedic materials (10), and pre-formed aggregates can later establish attached biofilms under shear (7). This dual behavior broadens the relevant anatomic niche from the implant surface to synovial fluid and adjacent infected tissue and may help explain why aspiration, debridement, and local antimicrobial delivery do not interrogate or reach every microbial compartment equally.

Although S. aureus is the mechanistic focus here, PJIs are microbiologically heterogeneous. In a United States series of 2,067 hip and knee PJI episodes, coagulase-negative staphylococci accounted for 37% of episodes and 25% were polymicrobial (15). In a 34-center Chinese cohort of 925 PJIs, S. aureus accounted for 26.5% and coagulase-negative staphylococci for 14.3%, with Gram-negative bacilli, anaerobes, mycobacteria, fungi, and polymicrobial infections also represented (16). Another Chinese series found Gram-positive cocci in 63.9% of isolates and coagulase-negative staphylococci in 38.1% (17). Clinically relevant biofilm-forming pathogens therefore include Staphylococcus epidermidis and other coagulase-negative staphylococci, S. lugdunensis, streptococci, enterococci, Cutibacterium acnes, and Gram-negative bacilli such as Pseudomonas aeruginosa and Escherichia coli. Their matrix chemistry, adhesins, intracellular behavior, and immune signaling differ, so mechanisms derived from S. aureus should be treated as a framework to test rather than assumed to be universal (15–17).

The implant itself further modifies this host-microbial interface. Adsorbed host proteins create a conditioning layer that provides microbial adhesion sites, while the foreign-body reaction alters vascularity, macrophage behavior, and extracellular matrix deposition (18). Recent work indicates that S. aureus extracellular adherence proteins can reduce biofilm porosity and protect bacteria from macrophage phagocytosis (19). Biofilm-secreted factors from S. aureus and Staphylococcus epidermidis also alter osteoblast differentiation in isolate- and patient-dependent ways (20). Thus, the pathogenic unit is not the bacterium alone, but the microorganism-matrix-implant-host interface.

## 3 Neutrophils: incomplete clearance and maladaptive activation

Neutrophils are prominent in acute and chronic implant infection, yet the interaction between neutrophils and biofilm is not an absolute phagocytic block. *In vitro* studies show that polymorphonuclear neutrophils can migrate into and phagocytose S. aureus biofilms, with the efficiency of this response influenced by biofilm maturation and opsonization (21, 22). At the same time, mature or structurally protected biofilm can remain incompletely cleared while neutrophils release proteases, reactive oxygen species, and neutrophil extracellular traps (NETs) (13, 14). A recent direct comparison of leukocyte responses to biofilm and planktonic S. aureus further demonstrated population-specific differences in biofilm-induced cytotoxicity, reinforcing that the biofilm state modifies rather than uniformly abolishes phagocyte function (23). Accordingly, “frustrated phagocytosis” is best viewed as a context-dependent outcome of restricted access and incomplete eradication, not as a universal inability of neutrophils to internalize biofilm-associated bacteria.

The clinical implication is that a neutrophil-rich aspirate or tissue section demonstrates an active host response but does not establish successful antimicrobial immunity. Neutrophil abundance should not be equated with effective microbial clearance; partial phagocytosis, persistent viable biofilm, NET formation, and collateral tissue injury may coexist. Any future strategy directed at this compartment would therefore need to preserve chemotaxis and bacterial killing while avoiding excessive degranulation, protease-mediated injury, and NET-associated matrix effects.

## 4 Macrophage reprogramming and defective bacterial killing

Macrophages normally integrate microbial recognition, phagocytosis, cytokine production, efferocytosis, and tissue repair. In implant biofilm, these functions become uncoupled. Some macrophages retain inflammatory signaling but have poor access to organisms, whereas others acquire IL-10-rich, repair-like programs that restrain bactericidal activity. In a murine S. aureus PJI model, biofilm-associated monocytes displayed a metabolic bias toward oxidative phosphorylation with reduced aerobic glycolysis; nanoparticle-mediated delivery of oligomycin shifted monocytes toward a pro-inflammatory metabolic state and improved clearance when combined with antibiotics (24). This establishes metabolic reprogramming as a mechanistic determinant of biofilm control in an experimental model, but not as a clinically validated intervention.

Biofilm metabolites can actively impose immunoregulatory programs on myeloid cells. Heim et al. showed that S. aureus biofilm-derived lactate inhibits HDAC11, permitting increased HDAC6 activity and histone H3 acetylation at the Il10 promoter, thereby enhancing IL-10 transcription in MDSCs and macrophages; the study combined *in vitro* systems with murine PJI and also reported elevated D-lactate and IL-10 in human PJI synovial fluid (25). This pathway provides direct mechanistic evidence that a bacterial metabolite can reprogram host epigenetic and cytokine responses during persistent biofilm infection.

The interaction is reciprocal. During experimental PJI, S. aureus formate metabolism is altered during contact with macrophages and G-MDSCs (26). At bacterial single-cell resolution, BaSSSh-seq further revealed extensive transcriptional heterogeneity in biofilm compared with planktonic growth and identified immune-cell-specific changes in bacterial metabolic, stress-response, and virulence programs after leukocyte exposure (27). Host responses are also conditioned by anatomical context: comparative transcriptomic and metabolomic analyses of experimental biofilm infection show that immune and metabolic signatures vary by tissue niche and evolve over time (28). Together, these findings support bidirectional immunometabolic crosstalk rather than a one-way model in which biofilm simply suppresses a static host response.

## 5 Granulocytic myeloid-derived suppressor cells and adaptive immune paralysis

Human evidence for G-MDSC enrichment should be separated from mechanistic evidence derived from experimental models. In a direct comparison of human PJI tissue with aseptic loosening, Heim et al. identified CD33-positive (CD33+) HLA-DR-negative (HLA-DR-) CD66b-positive (CD66b+) CD14-negative/low (CD14-/low) G-MDSCs as the predominant leukocyte population in PJI and reported reduced T-cell abundance together with increased local IL-10, IL-6, and CXCL1 (29). These data establish preferential G-MDSC accumulation in human PJI relative to aseptic tissue. More broadly, G-MDSCs can suppress effector responses through cytokine, arginase, reactive oxygen species (ROS), and metabolic mechanisms, although the relative contribution of each pathway is context dependent (30).

Mechanistic evidence for a glycolysis/HIF-1alpha program is strongest in murine PJI. Horn et al. showed that G-MDSCs in a mouse S. aureus PJI model display enhanced glycolysis and HIF-1alpha activity; nanoparticle delivery of 2-deoxy-D-glucose (2-DG) and granulocyte-targeted Hif1a deletion reduced suppressive activity and bacterial burden (31). The human component of that study comprised tissue and paired peripheral blood from three PJI patients and provided limited transcriptomic corroboration of glycolysis, hypoxia, and IL-10-associated signatures rather than a direct comparison with aseptic loosening (31). Separately, in a mouse model of chronic S. aureus infection, highly glycolytic MDSCs released lactate that impaired CD4+ T-cell lactate export, NAD+ regeneration, glycolytic flux, proliferation, and cytokine production (32). Thus, lactate-mediated metabolic suppression offers a plausible bridge from myeloid dysfunction to adaptive immune impairment, but its magnitude and causal importance in human PJI remain to be established. These evidence levels, candidate research readouts, and hypothesis-based intervention concepts are summarized in **Table 1**.

**Table 1:** Immune dysfunction nodes, candidate research readouts, and hypothesis-based intervention concepts in orthopedic implant biofilm.

Immune dysfunction node	Mechanistic consequence	Potential research readout	Hypothesis-based strategy	Main translational risk	References
Biofilm matrix exclusion	Limits phagocyte access and creates metabolic gradients	Matrix density, extracellular DNA, porosity, organism burden	Debridement; investigational matrix-degrading adjuncts; anti-adhesion surfaces	Dispersal of viable bacteria; tissue toxicity	(5–7, 10–12, 19)
Incomplete neutrophil clearance/maladaptive activation	Maturation-dependent phagocytosis may coexist with residual biofilm, degranulation, and NET-associated injury	Neutrophil enzymes, NET markers, viable biofilm after exposure	Preserve bacterial killing while limiting excessive NET/protease-mediated injury	Impaired early containment; tissue injury if over-suppressed or over-activated	(13, 14, 21–23)
Macrophage metabolic failure	Impaired biofilm access and bactericidal metabolic programming	Cell-state/metabolic profile; ex vivo biofilm killing	Local metabolic reprogramming combined with antimicrobial therapy	Off-target metabolic effects; cytokine injury and osteolysis	(24–28)
G-MDSC expansion	IL-10, arginase, ROS, and metabolic suppression of effector cells	CD33+HLA-DR-CD66b+CD14-/low cells; local IL-10/lactate; HIF-1alpha/glycolysis signature	Local, time-limited modulation of dominant suppressive pathways	Loss of inflammation control; lack of G-MDSC-specific targets	(25, 29–31)
CD4+ T-cell metabolic impairment	Lactate-associated restriction of NAD+ regeneration and glycolytic activation in experimental chronic S. aureus infection	T-cell proliferation/cytokines; metabolic flux; lactate handling	Reduce suppressive myeloid burden or restore local metabolic competence	Excessive inflammation or loss of immune regulation	(32)
Bone-immune uncoupling	Osteoclastogenesis, osteoblast injury, fibrosis, and poor perfusion	RANKL/OPG, vascular and bone-turnover markers	Sequence antimicrobial control before regenerative modulation	Premature repair signaling may preserve bacteria	(2, 14, 20)

The intervention concepts are investigational, require further validation, and should not be interpreted as clinical recommendations.

CD, cluster of differentiation; G-MDSC, granulocytic myeloid-derived suppressor cell; HDAC, histone deacetylase; HIF-1alpha, hypoxia-inducible factor 1-alpha; HLA-DR, human leukocyte antigen-DR; IL, interleukin; NAD+, oxidized nicotinamide adenine dinucleotide; NET, neutrophil extracellular trap; OPG, osteoprotegerin; PJI, periprosthetic joint infection; RANKL, receptor activator of nuclear factor-kappaB ligand; ROS, reactive oxygen species.

## 6 Bone-interface consequences

Persistent immune dysfunction directly affects reconstruction. Neutrophil products, inflammatory cytokines, and microbial toxins injure osteoblast-lineage cells and local vasculature (14, 20). Macrophage and stromal signals can increase receptor activator of nuclear factor-kappaB ligand (RANKL)-dependent osteoclastogenesis. Fibrotic encapsulation and abscess-associated stromal remodeling can reduce perfusion, further limiting antibiotic delivery and oxygen-dependent antimicrobial mechanisms. At the same time, excessive anti-inflammatory or pro-regenerative polarization may protect bone-forming cells while permitting bacterial survival.

This creates a repair-defense trade-off. The optimal immune state during infection is not a simple shift from pro-inflammatory to repair-associated macrophage states. Early treatment requires effective microbial killing; later phases require inflammation resolution and restoration of osteogenesis. Spatial control is equally important: strong antimicrobial activity at the biofilm interface should not cause diffuse damage to viable bone and soft tissue. This trade-off supports stage- and compartment-specific investigation rather than indiscriminate immune activation.

## 7 Therapeutic implications

Surgery and antibiotics remain the foundation of treatment because no host-directed intervention can compensate for an unstable implant, necrotic tissue, or mature biofilm burden that requires mechanical source control. The host-directed strategies discussed here remain preclinical. In murine PJI, monocyte-targeted oligomycin nanoparticles altered metabolic programming and improved biofilm clearance with antibiotics (24), whereas G-MDSC-directed 2-DG nanoparticles and granulocyte-targeted Hif1a deletion provided mechanistic proof that the glycolysis/HIF-1alpha axis is biologically tractable (31). However, these approaches face major translational barriers: glycolysis and HIF-1alpha are broadly used by neutrophils, macrophages, lymphocytes, osteoblast-lineage cells, and repair programs; current interventions are not intrinsically G-MDSC-specific; and genetic Hif1a deletion is a mechanistic tool rather than a deployable therapy. These experiments therefore establish biological tractability, not immediate therapeutic feasibility.

Other adjunctive approaches require the same evidentiary caution. Ticagrelor showed antibacterial and antibiofilm activity *in vitro* and reduced S. aureus implant burden in a mouse PJI model, but the investigators explicitly characterized the study as very preliminary with respect to clinical implications (33). Conversely, clinical translation has progressed further for direct biofilm targeting: a first-in-human phase 1 study evaluated the biofilm-disrupting monoclonal antibody TRL1068 in chronic hip or knee PJI (34). TRL1068 is directed at bacterial biofilm rather than host immunity and therefore should not be interpreted as evidence that host-directed immunotherapy is clinically established in PJI.

A biomarker-guided strategy is consequently a research priority rather than a current clinical algorithm. Candidate translational readouts include the proportion of CD33+HLA-DR-CD66b+CD14-/low G-MDSCs in synovial fluid or periprosthetic tissue (29), local lactate and IL-10 concentrations (25, 29), glycolysis/HIF-1alpha transcriptional signatures (31), and CD4+ T-cell proliferative, cytokine, or metabolic-flux measures (32). These measures have not been validated as diagnostic or treatment-selection biomarkers for PJI, and thresholds, temporal stability, organism specificity, and relationships to clinical outcome remain undefined. Accordingly, the proposal that future adjuncts should be local, time limited, and directed at a demonstrated dysfunction is a conceptual framework for hypothesis testing, not an evidence-based treatment recommendation. Any intervention would need to be integrated with source control and pathogen-directed antimicrobial therapy.

## 8 Conclusions

Biofilm-associated orthopedic infection should be understood as a disease of microbial persistence and host immune dysfunction shaped by a shared metabolic microenvironment. The matrix limits access, but persistence also reflects context-dependent neutrophil activity, macrophage metabolic reprogramming, G-MDSC accumulation, and adaptive immune suppression. At present, human PJI evidence primarily supports host-side associations—G-MDSC enrichment (29), increased local IL-10 (25, 29), and elevated synovial-fluid D-lactate (25)—whereas the bacterial adaptation/host-to-bacteria arm (26–28) and lactate-mediated suppression of CD4+ T-cell metabolism (32) remain supported by *in vitro* and/or animal models and have not yet been directly confirmed in human tissue. This distinction is essential when translating mechanism into therapy.

A testable translational framework is to combine source control and optimized antimicrobial treatment with precise correction of a demonstrated local dysfunction, while explicitly accounting for timing, tissue niche, and off-target immune or skeletal effects. Host-directed immunometabolic therapy for PJI remains preclinical, and current data do not support routine clinical use. Future studies should prioritize adequately powered human PJI cohorts, organism-resolved analyses, longitudinal sampling, and prospective validation of candidate metabolic and immune readouts to determine whether dysfunction is a cause of persistence, a consequence of chronic infection, or both.

## References

[1] Patel R . Periprosthetic joint infection. N Engl J Med. (2023) 388:251–62. doi: 10.1056/NEJMra2203477 36652356

[2] Masters EA Ricciardi BF Bentley KLM Moriarty TF Schwarz EM Muthukrishnan G . Skeletal infections: microbial pathogenesis, immunity and clinical management. Nat Rev Microbiol. (2022) 20:385–400. doi: 10.1038/s41579-022-00686-0 35169289 PMC8852989

[3] Sauer K Stoodley P Goeres DM Hall-Stoodley L Burmolle M Stewart PS . The biofilm life cycle: expanding the conceptual model of biofilm formation. Nat Rev Microbiol. (2022) 20:608–20. doi: 10.1038/s41579-022-00767-0 35922483 PMC9841534

[4] Azimi S Lewin GR Whiteley M . The biogeography of infection revisited. Nat Rev Microbiol. (2022) 20:579–92. doi: 10.1038/s41579-022-00683-3 35136217 PMC9357866

[5] Bidossi A Bottagisio M Savadori P De Vecchi E . Identification and characterization of planktonic biofilm-like aggregates in infected synovial fluids from joint infections. Front Microbiol. (2020) 11:1368. doi: 10.3389/fmicb.2020.01368 32714301 PMC7344156

[6] Dastgheyb S Parvizi J Shapiro IM Hickok NJ Otto M . Effect of biofilms on recalcitrance of staphylococcal joint infection to antibiotic treatment. J Infect Dis. (2015) 211:641–50. doi: 10.1093/infdis/jiu514 25214518 PMC4318921

[7] Gupta TT Gupta NK Burback P Stoodley P . Free-floating aggregate and single-cell-initiated biofilms of Staphylococcus aureus. Antibiotics (Basel). (2021) 10:889. doi: 10.3390/antibiotics10080889 34438938 PMC8388712

[8] Brandquist ND Kielian T . Immune dysfunction during S. aureus biofilm-associated implant infections: opportunities for novel therapeutic strategies. NPJ Biofilms Microbiomes. (2025) 11:144. doi: 10.1038/s41522-025-00782-y 40715142 PMC12297603

[9] Ogunware AE Kielian T . Immunometabolism shapes chronic Staphylococcus aureus infection: insights from biofilm infection models. Curr Opin Microbiol. (2025) 86:102612. doi: 10.1016/j.mib.2025.102612 40409167 PMC12276999

[10] Pestrak MJ Gupta TT Dusane DH Guzior DV Staats A Harro J . Investigation of synovial fluid induced Staphylococcus aureus aggregate development and its impact on surface attachment and biofilm formation. PloS One. (2020) 15:e0231791. doi: 10.1371/journal.pone.0231791 32302361 PMC7164621

[11] Ibberson CB Parlet CP Kwiecinski J Crosby HA Meyerholz DK Horswill AR . Hyaluronan modulation impacts Staphylococcus aureus biofilm infection. Infect Immun. (2016) 84:1917–29. doi: 10.1128/IAI.01418-15 27068096 PMC4907140

[12] Knott S Curry D Zhao N Metgud P Dastgheyb SS Purtill C . Staphylococcus aureus floating biofilm formation and phenotype in synovial fluid depends on albumin, fibrinogen, and hyaluronic acid. Front Microbiol. (2021) 12:655873. doi: 10.3389/fmicb.2021.655873 33995317 PMC8117011

[13] Versey Z da Cruz Nizer WS Russell E Zigic S DeZeeuw KG Marek JE . Biofilm-innate immune interface: contribution to chronic wound formation. Front Immunol. (2021) 12:648554. doi: 10.3389/fimmu.2021.648554 33897696 PMC8062706

[14] Bjarnsholt T Whiteley M Rumbaugh KP Stewart PS Jensen PO Frimodt-Moller N . The importance of understanding the infectious microenvironment. Lancet Infect Dis. (2022) 22:e88–92. doi: 10.1016/S1473-3099(21)00122-5 34506737 PMC9190128

[15] Tai DBG Patel R Abdel MP Berbari EF Tande AJ . Microbiology of hip and knee periprosthetic joint infections: a database study. Clin Microbiol Infect. (2022) 28:255–9. doi: 10.1016/j.cmi.2021.06.006 34129907 PMC8665939

[16] Peng HM Zhou ZK Wang F Yan SG Xu P Shang XF . Microbiology of periprosthetic hip and knee infections in surgically revised cases from 34 centers in mainland China. Infect Drug Resist. (2021) 14:2411–8. doi: 10.2147/IDR.S305205 34211286 PMC8241811

[17] Hu L Fu J Zhou Y Chai W Zhang G Hao L . Trends in microbiological profiles and antibiotic resistance in periprosthetic joint infections. J Int Med Res. (2021) 49:3000605211002784. doi: 10.1177/03000605211002784 33787371 PMC8020254

[18] Rosman CWK van Dijl JM Sjollema J . Interactions between the foreign body reaction and Staphylococcus aureus biomaterial-associated infection. Winning strategies in the derby on biomaterial implant surfaces. Crit Rev Microbiol. (2022) 48:624–40. doi: 10.1080/1040841X.2021.2011132 34879216

[19] Bhattacharya M Scherr TD Lister J Kielian T Horswill AR . Extracellular adherence proteins reduce matrix porosity and enhance Staphylococcus aureus biofilm survival during prosthetic joint infection. Infect Immun. (2025) 93:e0008625. doi: 10.1128/iai.00086-25 40116480 PMC11977312

[20] Tubel J Maier E Jegen M Marthen C Obermeier A Haug AT . Patient-specific effects of soluble factors from Staphylococcus aureus and Staphylococcus epidermidis biofilms on osteogenic differentiation of primary human osteoblasts. Sci Rep. (2021) 11:17282. doi: 10.1038/s41598-021-96719-4 34446785 PMC8390505

[21] Gunther F Wabnitz GH Stroh P Prior B Obst U Samstag Y . Host defence against Staphylococcus aureus biofilms infection: phagocytosis of biofilms by polymorphonuclear neutrophils (PMN). Mol Immunol. (2009) 46:1805–13. doi: 10.1016/j.molimm.2009.01.020 19261332

[22] Stroh P Gunther F Meyle E Prior B Wagner C Hansch GM . Host defence against Staphylococcus aureus biofilms by polymorphonuclear neutrophils: oxygen radical production but not phagocytosis depends on opsonisation with immunoglobulin G. Immunobiology. (2011) 216:351–7. doi: 10.1016/j.imbio.2010.07.009 20850891

[23] Brandquist ND Kielian T . Differential sensitivity of leukocyte populations to Staphylococcus aureus biofilm. Infect Immun. (2026) 94:e0065425. doi: 10.1128/iai.00654-25 41609497 PMC12974123

[24] Yamada KJ Heim CE Xi X Attri KS Wang D Zhang W . Monocyte metabolic reprogramming promotes pro-inflammatory activity and Staphylococcus aureus biofilm clearance. PloS Pathog. (2020) 16:e1008354. doi: 10.1371/journal.ppat.1008354 32142554 PMC7080272

[25] Heim CE Bosch ME Yamada KJ Aldrich AL Chaudhari SS Klinkebiel D . Lactate production by Staphylococcus aureus biofilm inhibits HDAC11 to reprogramme the host immune response during persistent infection. Nat Microbiol. (2020) 5:1271–84. doi: 10.1038/s41564-020-0756-3 32661313 PMC7529909

[26] Bertrand BP Heim CE West SC Chaudhari SS Ali H Thomas VC . Role of Staphylococcus aureus formate metabolism during prosthetic joint infection. Infect Immun. (2022) 90:e0042822. doi: 10.1128/iai.00428-22 36286525 PMC9670962

[27] Korshoj LE Kielian T . Bacterial single-cell RNA sequencing captures biofilm transcriptional heterogeneity and differential responses to immune pressure. Nat Commun. (2024) 15:10184. doi: 10.1038/s41467-024-54581-8 39580490 PMC11585574

[28] Van Roy Z Arumugam P Bertrand BP Shinde DD Thomas VC Kielian T . Tissue niche influences immune and metabolic profiles to Staphylococcus aureus biofilm infection. Nat Commun. (2024) 15:8965. doi: 10.1038/s41467-024-53353-8 39420209 PMC11487069

[29] Heim CE Vidlak D Odvody J Hartman CW Garvin KL Kielian T . Human prosthetic joint infections are associated with myeloid-derived suppressor cells (MDSCs): implications for infection persistence. J Orthop Res. (2018) 36:1605–13. doi: 10.1002/jor.23806 29139571 PMC5953848

[30] Veglia F Sanseviero E Gabrilovich DI . Myeloid-derived suppressor cells in the era of increasing myeloid cell diversity. Nat Rev Immunol. (2021) 21:485–98. doi: 10.1038/s41577-020-00490-y 33526920 PMC7849958

[31] Horn CM Arumugam P Van Roy Z Heim CE Fallet RW Bertrand BP . Granulocytic myeloid-derived suppressor cell activity during biofilm infection is regulated by a glycolysis/HIF1a axis. J Clin Invest. (2024) 134:e174051. doi: 10.1172/JCI174051 38421730 PMC11014666

[32] Goldmann O Medina E . Myeloid-derived suppressor cells impair CD4+ T cell responses during chronic Staphylococcus aureus infection via lactate metabolism. Cell Mol Life Sci. (2023) 80:221. doi: 10.1007/s00018-023-04875-9 37480485 PMC10363054

[33] Pant N Miranda-Hernandez S Rush C Warner J Eisen DP . Non-antimicrobial adjuvant therapy using ticagrelor reduced biofilm-related Staphylococcus aureus prosthetic joint infection. Front Pharmacol. (2022) 13:927783. doi: 10.3389/fphar.2022.927783 35846990 PMC9284533

[34] Conway J Delanois RE Mont MA Stavrakis A McPherson E Stolarski E . Phase 1 study of the pharmacokinetics and clinical proof-of-concept activity of a biofilm-disrupting human monoclonal antibody in patients with chronic prosthetic joint infection of the knee or hip. Antimicrob Agents Chemother. (2024) 68:e0065524. doi: 10.1128/aac.00655-24 39012102 PMC11304715

